# Comparative Chromosomal Localization of 45S and 5S rDNA Sites in 76 Purple-Fleshed Sweet Potato Cultivars

**DOI:** 10.3390/plants9070865

**Published:** 2020-07-08

**Authors:** Dan Su, Lei Chen, Jianying Sun, Luyue Zhang, Runfei Gao, Qiang Li, Yonghua Han, Zongyun Li

**Affiliations:** 1 Institute of Integrative Plant Biology, Jiangsu Key Laboratory of Phylogenomics & Comparative Genomics, School of Life Science, Jiangsu Normal University, Xuzhou 221100, China; dandan.SU@hotmail.com (D.S.); jsnuchenl@163.com (L.C.); jianyingsun@jsnu.edu.cn (J.S.); zly951004@163.com (L.Z.); 2 Jiangsu Xuhuai Regional Xuzhou Institute of Agricultural Sciences/Sweetpotato Research Institute, Chinese Academy of Agricultural Sciences, Xuzhou 221100, China; grf15852141027@gmail.com (R.G.); instrong@163.com (Q.L.)

**Keywords:** purple-fleshed sweet potato, fluorescence in situ hybridization, rDNA

## Abstract

In recent years, the purple-fleshed sweet potato has attracted more attention because of its high nutritional value. The cytogenetics of this crop is relatively unexplored, limiting our knowledge on its genetic diversity. Therefore, we conducted cytogenetic analysis of 76 purple-fleshed sweet potato cultivars to analyze the chromosome structure and distribution of 45S and 5S rDNA. We noted that only 62 cultivars had 90 chromosomes, and the others were aneuploid with 88, 89, 91, or 92 chromosomes. The number of 45S rDNA in the 76 cultivars varied from 16 to 21; these sites showed different signal sizes and intensities and were localized at the chromosomal termini or satellite. The number of 5S rDNA was relatively stable; 74 cultivars showed six sites located at the chromosomal sub-terminal or near the centromere. Only the ‘Quanzishu 96’ and ‘Yuzixiang 10’ showed seven and five 5S rDNA sites, respectively. Additionally, both parent cultivars of ‘Quanzishu 96’ showed 18 45S and six 5S rDNA sites. Overall, our results indicate a moderate diversity in the distribution pattern of rDNAs. Our findings provide comprehensive cytogenetic information for the identification of sweet potato chromosomes, which can be useful for developing a high-quality germplasm resource.

## 1. Introduction

The sweet potato (*Ipomoea batatas* (L.) Lam., 2n = 6x = 90), which belongs to the genus *Ipomoea* of the family Convolvulaceae, is the seventh most important food crop in the world. It has been widely distributed over tropical, subtropical, and warm temperate regions. China is the largest producer of sweet potatoes, with an annual production of about 72 million tons, accounting for 63.84% of the total production in the world [1].

The purple-fleshed sweet potato, a special type of sweet potato, has gradually developed into a cash crop and medicinal crop besides being a food crop [2]. It is rich in vitamins, dietary fiber, anthocyanins, and carotenoids [3,4,5]. The identification of the free radical-scavenging activity of these functional components has attracted the interest of health-conscious consumers. This activity is related to slowing of aging process and preventing chronic degenerative diseases such as cancer [6,7,8].

Currently, the research on purple-fleshed sweet potato is mainly focused on the extraction and purification of anthocyanin pigment, its physiological functions, and the production of anthocyanin-related products; moreover, researchers have made some progress in this regard [9,10,11]. However, due to the large number of extremely small chromosomes and thick cytoplasm, which are difficult to remove, the research on cytogenetics of purple-fleshed sweet potato is in its infancy. Although there are a few studies in this domain, their findings are insufficient to drive the development of high-quality purple-fleshed sweet potato cultivars. Therefore, the cytogenetic analysis of purple-fleshed sweet potato is particularly urgent [12].

Fluorescence in situ hybridization (FISH) is a relatively mature molecular cytogenetic technique that focuses on the study of phylogeny at the chromosomal level. FISH can not only identify the position of a target sequence but also perform its qualitative and relative quantitative analysis by combining labeled nucleic acid probes with chromosomes, interphase nucleus, or DNA fibers. It has been widely used for identifying specific chromosome regions, analyzing their composition, spatial location, and dynamic chromatin changes during the cell cycle [13,14,15]. Additionally, it has been widely used in unraveling the physical map, structure, and evolution of the genome and in analyzing the relationship between species [16,17,18,19,20,21].

Ribosomal DNA (rDNA) is the most widely used marker in evolutionary studies, which is comprised of 45S rDNA and 5S rDNA, and has shown to be a good cytogenetic marker [22]. In the genomes of most organisms, the 5S and 45S are tandemly arranged and presented in high copy number with different chromosomal distributions [23,24,25,26]. The 45S rDNA is located in the nucleolar organizer region and consists of tandem repetitive units of the 18S-5.8S-28S rRNA genes and non-transcribed spacer (NTS) regions. On the contrary, the 5S rDNA comprises a highly conserved coding region of 120 bp and a non-transcribed spacer (NTS) region that varies between 100 and 900 bp. These rDNA units are relatively stable and reliable molecular cytogenetic markers and provide useful information for chromosome research [24,25,26,27,28]. To date, 1791 species from 86 plant families have been analysed to provide information on rDNA number and distribution [26]. FISH with rDNA as probes has been used for discovering genome restructuring [29], intra-species genetic diversity [30], and re-discovering the status of certain species as well [31]. In the past few decades, some studies have used FISH to analyze the distribution and organization of 5S and 45S rDNA units of hexaploid *Ipomoea batatas*; however, they detected different 45S rDNA sites and six 5S rDNA sites [32,33,34,35].

In previous studies, the genetic diversity of 76 purple-fleshed sweet potato cultivars has been analyzed at the molecular level and morphological and qualitative characteristics of their genomes have been unraveled [36]. These studies have helped in understanding the differences among different cultivars and provided guidance in selecting parent cultivars for breeding programs. In this study, we conducted FISH experiments with 45S and 5S rDNA probes on metaphase spreads of 76 purple potato cultivars to investigate the numbers and physical positions of the rDNA units. The analysis of chromosome structure and 45S/5S rDNA distribution is of great significance for further understanding of the chromosomal relationships and can provide a cytological basis for studying the related germplasm resources of sweet potato.

## 2. Results

**Figure 1:**
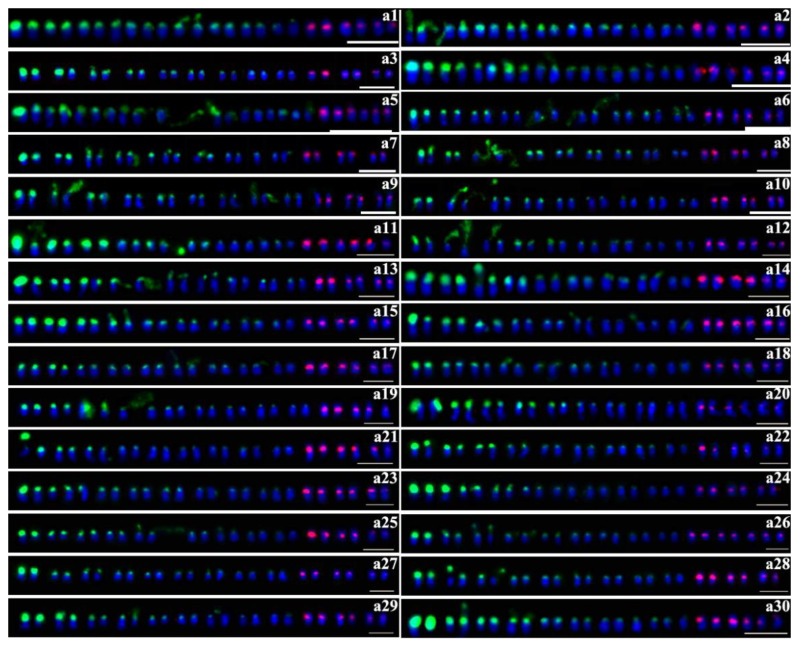
Distribution of 45S (green) and 5S (red) rDNA sites detected by FISH (fluorescence in situ hybridization) with the number of 45S rDNA is 18. a1: Neiyuzi 2; a2: Qinzishu 2; a3: Longzishu 6; a4: Zhezishu 1; a5: Jizishu 2; a6: Fuzishu 1; a7: Taizhong 11; a8: Yanzishu 3; a9: Jizishu 1; a10: Jizishu2; a11: Ningzishu 1; a12: Luozishu 1; a13: Xuzishu 8; a14: Yanzishu 4; a15: Guiziwei 1; a16: Chuanzishu 4; a17: Guijingshu 8; a18: Chuanzishu 2; a19: Yuzixiang 10; a20: Xuzishu 3; a21: Yuzishu 3; a22: Xuzishu 5; a23: Guijingshu 7; a24: Guijingshu 3; a25: Nanzishu 008; a26: Quanzishu 96; a27: Puzishu 18; a28: Xushu 33; a29: Jizishu 3; a30: Ayamurasaki. Scale bars, 5 μm.

**Figure 2:**
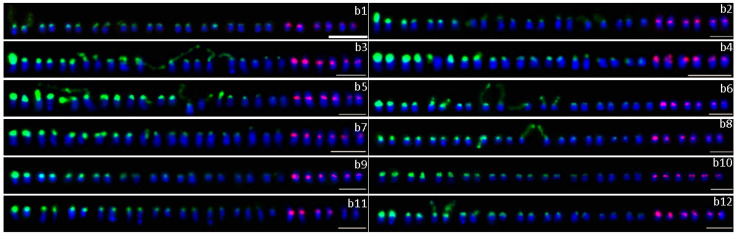
Distribution of 45S (green) and 5S (red) rDNA sites detected by FISH with the number of 45S rDNA is 20. b1: Wan W36-1; b2: Zhanzishu 2; b3: Mianzishu 9; b4: Mianyuzi 11; b5: Ezishu 13; b6: Fushu 404; b7: Fangzishu 9; b8: Yuzishu 7; b9: Pengzishu 1; b10: Funingzi 4; b11: Guangzishu 9; b12: Guangzishu 8. Scale bars, 5 μm.

**Figure 3:**
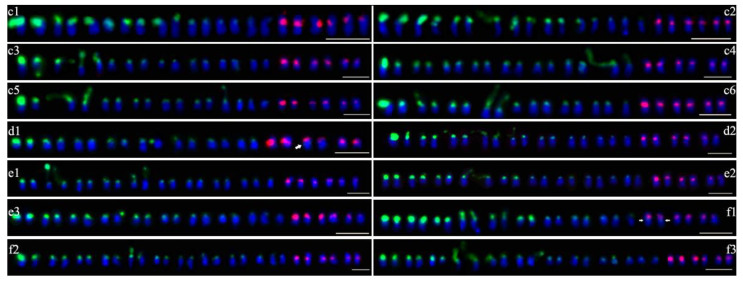
Distribution of 45S (green) and 5S (red) rDNA sites detected by FISH with the number of 45S rDNA is 16,17,19,21. c1–c6: the number of 45S rDNA is 16, c1: Ningzishu 2; c2: Guizishu 1; c3: Yusuzi 43; c4: Guijingshu 6; c5: Nanzishu 014; c6: Nanzishu 015. d1–d2: the number of 45S rDNA is 17, d1: Ezishu 12, the colocalization site is shown by arrowhead; d2: Qianzishu 1. e1–e3: the number of 45S rDNA is 19, e1: Yanzishu 2; e2: Puzishu 3; e3: Guangzishu 11. f1–f3: the number of 45S rDNA is 21, f1: Ningzishu 4, the colocalization sites are shown by arrowheads; f2: Funingzi 3; f3: Jizishu 18. Scale bars, 5 μm.

**Figure 4:**
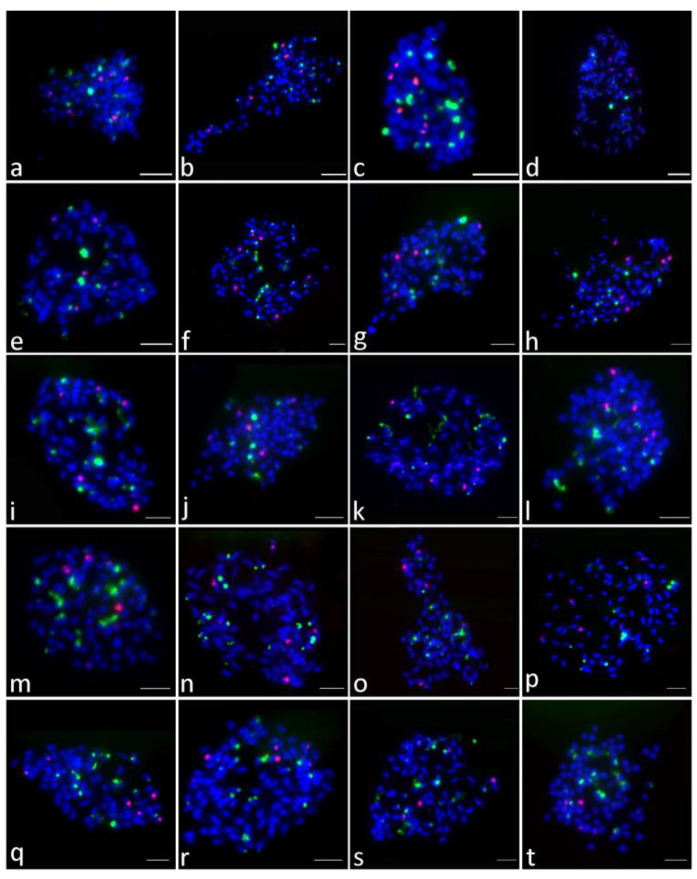
Distribution of 45S (green) and 5S (red) rDNA sites detected by FISH in the remain 20 purple sweet potato cultivars. a: Quzishu 57; b: Longzishu 4; c: Qinzishu 3; d: Ningzishu 3; e: Longjinshu 1; f: Longjinshu 3; g: Xuzishu 2; h: Ningzishu 5; i: Xuzishu 6; j: Jizishu 1; k: Wanzi 56; l: Guizishu 3; m: Fushu 24; n: Guijingshu 9; o: Shangxuzi 1; p: Jixuzi 2; q: Yuzi 263; r: Guangzishu 10; s: Guangzishu 1; t: Guangzishu 2. Scale bars, 5 μm.

Seventy-six purple-fleshed sweet potato cultivars were analyzed in this study. Double-color FISH was used to investigate the distribution and site numbers of 45S and 5S rDNA units. The patterns with well-spread chromosomes and distinguishable FISH signals were used for the analysis. At least 20 metaphase or prophase spreads were studied to generate the FISH results for each cultivar. The number and intensity of rDNA sites of 76 cultivars are summarized in Table 1. The number and chromosomal positions of 45S and 5S rDNA sites of 76 cultivars are presented in Figure 1, Figure 2, Figure 3 and Figure 4.

### 2.1. Chromosome Numbers

Among the 76 purple-fleshed sweet potato cultivars studied here, the chromosome number of 62 cultivars was 90. Out of the other 14 cultivars, four had 88 chromosomes, five had 89, three had 91, and two had 92 (Table 1, Figure S1).

### 2.2. Number of 45S rDNA and 5S rDNA Sites

In the 76 purple-fleshed sweet potato cultivars, the number of 45S rDNA sites was different. Overall, 43 cultivars showed 18 45S rDNA sites (the results of 30 cultivars are shown in Figure 1), 18 cultivars showed 20 (12 are shown in Figure 2), seven cultivars showed 16 (six are shown in Figure 3, c1–c6), two cultivars showed 17 (Figure 3, d1–d2), three cultivars showed 19 (Figure 3, e1–e3), and the other three cultivars showed 21 (Figure 3, f1–f3). The results of the other 20 cultivars are shown in Figure 4. More FISH results of sweet potato cultivars with the number of 45S rDNA is 16, 17, 19, 20, 21 had provided in the supplementary file, Figure S2.

Compared to that of 45S rDNA, the number of 5S rDNA sites was conserved across 76 cultivars—except for two. Among the 76 cultivars studied here, 74 cultivars showed six 5S rDNA sites (Figure 1, Figure 2, Figure 3 and Figure 4, Table 1), except for Quanzishu 96 and Yuzixiang 10. Seven sites of 5S rDNA were detected in Quanzishu 96 (Figure 1, a26; Figure S3) and five sites were detected in Yuzixiang 10 (Figure 1, a19; Figure S3).

Based on the presence of seven 5S rDNA sites in Quanzishu 96, a double-color FISH mapping of 45S and 5S rDNA was carried out for its two parent cultivars (Longshu 9 and Quanshu 10). However, Longshu 9 and Quanshu 10 showed 18 45S rDNA and six 5S rDNA hybridization signals, which was not similar to the signal pattern observed in the case of Quanzishu 96 (data not shown).

### 2.3. Distribution of 45S rDNA and 5S rDNA Sites

FISH results indicated that 45S rDNA sites were mainly located at chromosomal termini and satellite chromosomes. The signal intensity of different cultivars showed significant differences, and the signal intensity of different chromosomes of the same cultivar also has great differences (Table 1, Figure 1, Figure 2, Figure 3 and Figure 4).

5S rDNA sites were mainly located at the sub-terminal regions of chromosomes and near centromere. The 5S rDNA site size and intensity were different between cultivars and within individual cultivars (Table 1, Figure 1, Figure 2, Figure 3 and Figure 4). 

### 2.4. Colocalization of 45S and 5S rDNA Sites

Colocalization of 45S and 5S rDNA sites was investigated based on the FISH signals in 76 cultivars. Only two cultivars showed colocalization of 45S and 5S rDNA sites in the same chromosome (Figure 3, d1, f1, arrows).

## 3. Discussion

rDNA is highly repetitive and conserved across various species. In the case of plant species with a large number of small-sized chromosomes, the number and distribution of rDNA sites revealed by FISH probes provides a cytological approach for studying interspecies genetic relationships. The distribution pattern of rDNA sites is generally different in different species. Observing the differences in the number and distribution pattern of rDNA sites can help in further analyzing the chromosomal behavior of species from different genera [37]. Considering this, the number and distribution pattern of rDNA sites is a relatively reliable and stable molecular cytogenetic marker.

In this study, we conducted a comprehensive statistical analysis of the chromosome numbers and 45S/5S rDNA distribution patterns of 76 purple-fleshed sweet potato cultivars. We found that only 62 cultivars had 90 chromosomes as expected, and the others were aneuploid with 88, 89, 91, or 92 chromosomes (Table 1, Figure S1). Wu et al. [38] have used a statistical test to detect 6× + 1 and 6× − 2 aneuploidy in sweet potato cultivars based on read depth and showed that aneuploidy might present an extreme form of structural variation that affects transcript dosage and consequently changes phenotypic variation. Similar studies in potato have also revealed extensive structural variations, including presence/absence variation of sequences up to 575 kb in length, impacting transcript dosage [39,40,41].

In our study, the number of 45S rDNA sites was variable in hexaploid cultivars. In previous studies, researchers have found that the number of 45S rDNA sites varies from 12 to 22 and the number of 5S rDNA sites is six [32,33,34,35]. Over the past few decades, researchers have found that the intraspecific variation in the number and intensity of 45S rDNA signals is common; further, the distribution patterns of rDNA sites often vary between closely related species, and these patterns have been confirmed to be highly variable and unstable in many species [30,42,43,44,45]. Compared with previous studies, our results confirmed the high variability in the number of 45S rDNA sites across the studied cultivars, ranging from 16 to 21 (Figure 1, Figure 2, Figure 3 and Figure 4, Figure S2; Table 1). Excluding the influence of objective factors, the intraspecific variation in the number and distribution pattern of rDNA sites may be attributed to three mechanisms: unequal crossing over and transposition event, chromosomal structure fracture and rearrangement, and polyploidy process changes to different degrees. Owing to transposon activity, the intra-genome migration of rRNA genes has been widely reported in seed plants, and this is speculated to be one of the main factors driving the evolution of rDNA sites. Schubert et al. [46] have shown that the entire 45S rDNA repeat sequence in the chromosomes of Allium and its subgenus can be freely transferred from one site to another, indicating that 45S rDNA may move as a transposable element. Recently, the 45S rDNA region has been shown to be a fragile site (brittle site) prone to chromosomal damage in several species [47]. The increase in the number of 45S rDNA sites above a critical threshold also increases the possibility of chromosomal breakage in the rDNA region [48]. Thomas et al. [49] have shown that 45S rDNA cleavage in rye grass leads to the rearrangement of chromosome structure, and the position and number of 45S rDNA sites were different. The third mechanism of changes in the polyploidy process to different degrees has been widely reported [50,51,52]. Srisuwan et al. [32] considered that the interspecific and intraspecific variation in the number of 45S rDNA sites in hexaploid *I. batatas* might be because the hexaploid genome of *I. batatas* is unstable and always in the process of diploidization. In addition, 45S rDNA is mainly located at the chromosomal termini and on the satellite chromosomes, which may contribute to the variable number of 45S rDNA sites. As 45S rDNA sites often break off from the chromosomal termini to the satellite chromosomes, it might be more prone to unequal crossing over or ectopic recombination. Mantovani et al. [53] also considered that a large number of hybridization events during cultivation may be the cause of rDNA site changes.

According to previous studies [32,33,34,35], the number of 5S rDNA sites in cultivated sweet potato is relatively stable, and six 5S rDNA sites have been detected. In this study, among the 76 purple-fleshed sweet potato cultivars, the number of 5S rDNA sites was six in 74 cultivars, and it was different in the other two cultivars: seven in Quanzishu 96 (Figure 1, a26; Figure S3) and five in Yuzixiang 10 (Figure 1, a19; Figure S3). However, Longshu 9 and Quanshu 10, which were parent cultivars of Quanzishu 96, showed 18 45S rDNA sites and six 5S rDNA hybridization signals, which was counterintuitive. Some studies have suggested that the increase in the number of 5S rDNA sites may be caused by the amplification of the covert rDNA copy number during crossing over or transposition events [54,55]. It could also be because of the translocation of rDNA genes to chromosomes without rDNA sites. On the contrary, the decrease in the number of rDNA sites may be caused by fusion with other DNA sequences [55]. 

The signal size and intensity of 45S and 5S rDNA sites are different in different cultivars. Moreover, within the same cultivar, the intensity of the signal is positively correlated to the copy number, which means that a weak signal indicates a relatively low copy number. Events such as amplification, deletion, and unequal crossing over can also affect the copy number and result in signal differences.

In general, the evolution of 45S and 5S rDNA is independent of each other and these sites tend to be distributed on different chromosomes due to physical distance [56]. Among the cultivars we studied, two cultivars showed adjacent localization of 5S and 45S rDNA sites. According to a study by Sun [57], colocalization was also observed in three cultivars of sweet potato. Roa and Guerra [58,59] believed that 5S–45S colocalization exists in at least one species of each genus, although the probability of colocalization is relatively low, as reported in the genera *Hordeum*, *Cucumis*, and *Brassica* [13,60,61]. Most of the adjacent 5S–45s sites were found located on the short arms of chromosomes. Due to the limited physical space of the short arm and the size of the FISH signal amplified by different methods, these sites may be treated as adjacent sites [59]. Zhang et al. [13] believed that, to some extent, the synapomorphy of 5S–45S rDNA linkage found in *Cucumis* is related to the geographical area. Roa and Guerra [59] believed that the frequency of linkage for 5S-45S is directly influenced by the number of sites per karyotype and other factors. Therefore, the occurrence of adjacent localization of 5S and 45S rDNA sites may be the result of the interaction between rDNA instability (the number of rDNA sites) and other unknown factors.

## 4. Materials and Methods 

### 4.1. Plant Materials

The 76 purple-fleshed sweet potato cultivars analyzed in this study were collected from the Institute of Sweet potato, Chinese Academy of Agricultural Sciences. The test cultivars Longshu 9 and Quanshu 10 (the parent cultivars of Quanzishu 96) were provided by the Institute of Agricultural Sciences of Quanzhou.

### 4.2. Chromosome Preparation

The mitotic chromosome preparation was performed using a protocol published previously [62] with minor modifications. The root tips were induced by cutting away the tip of the main root. Root tips of about 1 to 2 cm were pretreated with 2 mM 8-hydroxyquinoline at room temperature for 2 to 4 h in the dark to obtain dividing cells in metaphase. The root tips were then fixed in the Carnoy’s fixative solution (ethanol/acetic acid, 3:1, v/v) for at least one day. Subsequently, the root tips were thoroughly cleaned using deionized water and digested with a mixture of 2% cellulase and 1% pectinase for 2.5 h at 37 °C in a water bath. The enzyme solution was replaced by deionized water for 30 min. The slides were prepared using a “flame-dry” method: the root tips were first transferred on to a slide, mashed, and flame dried.

### 4.3. Probe Preparation

The 5S rDNA oligonucleotide probes 5S-1 and 5S-2 were the 1–59 and 60–118 base sequences of the 5S rRNA coding region of *Arabidopsis thaliana* (L) Heynhold, respectively (Table 2). The 45S rDNA oligonucleotide probes 45S-1, 45S-2, and 45S-3 were derived from partial sequences of the 5.8S, 18S, and 25S rRNA coding regions of *A. thaliana*, respectively. 5S-1 and 5S-2 were labeled with 6-carboxyl-tetramethyl rhodamine (TAMRA) at the 5′-terminus and then mixed together to make the 5S rDNA probe solution. 45S-1, 45S-2, and 45S-3 were labeled with 6-carboxyl fluorescein (6-FAM) at the 5′-terminus and mixed together to make a 45S rDNA probe solution. These oligonucleotide probes were synthesized by Sangon Bioengineering Co., LTD (Shanghai, China).

### 4.4. FISH and Signal Detection

FISH was performed according to the method described by Jiang et al. [63] with slight modifications. The hybridization solution was as follows: deionized formamide, 10 μL; 50% dextran sulphate, 4 μL; 20×SSC, 2 μL; salmon sperm DNA, 2 μL (40 ng); each probe DNA, 1 μL (40 ng). The slides were baked at 65 °C for 45 min, cooled, and then denatured in 70% deionized formamide at 85 °C for 2.5 min. Further, they were dehydrated in 70%, 90%, and 100% alcohol for 5 min at −20 °C and then dried in the air. The hybridization solution was applied to the pretreated chromosome slide at 20 μL and then incubated at 37 °C overnight.

The hybridized slides were eluted with 2×SSC and 1×TNT on a shaker. The chromosomes were counterstained with 4′,6-diamidino-2-phenylindole (DAPI) in the VectaShield antifade solution. The photographs were taken using the Leica DM6000B fluorescence microscope (Leica Camera AG, Wetzlar, Germany) or Olympus BX63 epifluorescence microscope (Olympus, Tokyo, Japan), and Adobe Photoshop CS 8.0 (Adobe Systems Incorporated, San Jose, California, USA) was used for adjusting image contrast.

## 5. Conclusions

We studied the molecular cytogenetics of 76 purple-fleshed sweet potato cultivars using rDNA–FISH. We noted that among hexaploid sweet potato cultivars, the purple-fleshed sweet potato cultivars show genetic instability. Overall, the 45S rDNA sites showed numerical variation, whereas the 5S rDNA sites were conserved in number. Our analysis provides comprehensive cytological information for the identification of sweet potato chromosomes. Additionally, it provides a cytological basis for the development of high quality purple-fleshed sweet potato germplasm resources.

## Figures and Tables

**Table 1:** Summary of rDNA results of 76 purple-fleshed sweet potato varieties.

No.	Cultivars	Chromosome Number (2n)	No. of 45S rDNA	No. of 5S rDNA	No.	Cultivars	Chromosome Number (2n)	No. of 45S rDNA	No. of 5S rDNA
1	Quzishu 57	90	18(1S,12M,5W)	6	39	Chuanzishu 4	90	18(1S,11M,6W)	6
2	Neiyuzi 2	92	18(6S, 8M,4W)	6	40	Fushu 24	90	20(4S,8M,8W)	6
3	Qinzishu 2	90	18(3S,11M,4W)	6	41	Guijingshu 8	90	18(3S,11M,4W)	6
4	Longzishu 4	90	18(6S,10M,2W)	6	42	Chuanzishu 2	88	18(2S,12M,4W)	6
5	Longzishu 6	90	18(3S,13M,2W)	6	43	Guijingshu 9	90	18(4S,14M)	6
6	Zhezishu 1	90	18(4S,12M,2W)	6	44	Fangzishu 9	90	20(2S,16M,2W)	6
7	Qinzishu 3	90	18(3S,13M,2W)	6	45	Shangxuzi 1	90	20(3S,11M,6W)	6
8	Ningzishu 3	90	18(2S,6M,10W)	6	46	Jixuzi 2	90	16(4S,9M,3W)	6
9	Jizishu 2	90	18(1S,13M,4W)	6	47	Yuzixiang 10	90	18(4S,6M,8W)	5
10	Longjinshu 1	90	18(2S,9M,7W)	6	48	Xuzishu 3	90	18(2S,10M,6W)	6
11	Fuzishu 1	90	18(2S,13M,3W)	6	49	Yusuzi 43	90	16(1S,11M,4W)	6
12	Taizhong 11	90	18(1S,12M,5W)	6	50	Yuzishu 7	90	20(2S,14M,4W)	6
13	Yanzishu 3	89	18(1S,13M,4W)	6	51	Pengzishu 1	90	20(2S,11M,7W)	6
14	Jizishu 1	90	18(3S,12M,3W)	6	52	Yuzishu 3	90	18(2S,8M,8W)	6
15	Wan W36-1	90	20(16M,4W)	6	53	Yuzi 263	88	18(2S,12M,4W)	6
16	Longjinshu 3	90	18(2S,14M,2W)	6	54	Xuzishu 5	90	18(2S,14M,2W)	6
17	Zhanzishu 2	90	20(3S,14M,3W)	6	55	Guijingshu 6	90	16(1S,14M,1W)	6
18	Jizishu 2	90	18(2S,14M,2W)	6	56	Guijingshu 7	90	18(1S,10M,7W)	6
19	Yanzishu 2	90	19(2S,12M,5W)	6	57	Guijingshu 3	90	18(3S,7M,8W)	6
20	Ningzishu 4	90	21(4S,12M,5W)	6	58	Nanzishu 008	91	18(2S,10M,6W)	6
21	Ningzishu 1	88	18(4S,10M,4W)	6	59	Nanzishu 014	90	16(1S,13M,2W)	6
22	Xuzishu 2	88	18(1S,13M,4W)	6	60	Nanzishu 015	90	16(1S,14M,1W)	6
23	Ningzishu 2	90	16(2S,10M,4W)	6	61	Qianzishu 1	91	17(1S,13M,3W)	6
24	Ningzishu 5	89	18(2S,11M,5W)	6	62	Funingzi 3	90	21(1S,18M,2W)	6
25	Luozishu 1	90	18(1S,11M,6W)	6	63	Funingzi 4	90	20(4S,11M,5W)	6
26	Xuzishu 8	90	18(6S,8M,4W)	6	64	Quanzishu 96	90	18(2S,12M,4W)	7
27	Xuzishu 6	90	20(1S,12M,7W)	6	65	Puzishu 3	90	19(4S,8M,7W)	6
28	Yanzishu 4	90	18(4S,12M,2W)	6	66	Puzishu 18	89	18(2S,13M,3W)	6
29	Jizishu 1	90	18(3S,9M,6W)	6	67	Xushu33	91	18(1S,13M,4W)	6
30	Guizishu 1	90	16(3S,9M,4W)	6	68	Jizishu 3	90	18(2S,12M,4W)	6
31	Mianzishu 9	90	20(1S,16M,3W)	6	69	Guangzishu 9	90	20(2S,12M,6W)	6
32	Mianyuzi 11	90	20(4S,12M,4W)	6	70	Guangzishu 10	90	18(1S,14M,3W)	6
33	Wanzi 56	90	18(2S,13M,3W)	6	71	Guangzishu 11	89	19(2S,11M,6W)	6
34	Ezishu 13	90	20(6S,11M,3W)	6	72	Guangzishu 1	90	20(2S,15M,3W)	6
35	Ezishu 12	89	17(2S,12M,3W)	6	73	Guangzishu 2	90	20(2S,16M,2W)	6
36	Guizishu 3	90	20(2S,15M,3W)	6	74	Guangzishu 8	90	20(2S,13M,5W)	6
37	Guiziwei 1	90	18(5S,12M,1W)	6	75	Jizishu 18	90	21(1S,17M,3W)	6
38	Fushu 404	92	20(2S,12M,6W)	6	76	Ayamurasaki	90	18(2S,14M,2W)	6

S: strong signal; M: moderate signal; W: weak signal.

**Table 2:** Name, sequence, and sources of oligonucleotide probes for fluorescence in situ hybridization (FISH) analysis.

Probe Name	Sequence and Fluorochrome Label	Sequences Used to Develop Probes (GenBank Accession Number)
5S-1	TAMRA-5′GGATGCGATCATACCAGCACTAAT GCACCGGATCCCATCAGAACTCCGCAGTTAA GCGT3′	1–59 bases in coding region of 5S rRNA from *Arabidopsis thaliana* (GenBank AJ307346.2)
5S-2	TAMRA-5′GCTTGGGCGAGAGTAGTACTAGGA TGGGTGACCTCTCGGGAAATCCTCGTGTTGC ATC3′	60–118 bases in coding region of 5S rRNA from *Arabidopsis thaliana* (GenBank AJ307346.2)
45S-1	6-FAM -5′AAAACGACTCTCGGCAACGGATAT CTCGGCTCTCGCATCGATGAAGAACGTAGCG AAAT3′	Coding region of 5.8S rRNA from *Arabidopsis thaliana* (GenBank NR141643.1)
45S-2	6-FAM -5′TACCTGGTTGATCCTGCCAGTAGTC ATATGCTTGTCTCAAAGATTAAGCCATGCAT GTG3′	Coding region of 18S rRNA from *Arabidopsis thaliana* (GenBank NR141642.1)
45S-3	6-FAM-5′CCCGCTGAGTTTAAGCATATCAATA AGCGGAGGAAAAGAAACTAACAAGGATTC CCTTA3′	Coding region of 25S rRNA from *Arabidopsis thaliana* (GenBank X52320.1)

## Supplementary Materials

The following are available online at https://www.mdpi.com/2223-7747/9/7/865/s1, Figure S1: Metaphase cells of aneuploids. Figure S2: Metaphase cells of sweet potato cultivars with the number of 45S rDNA is 16, 17, 19, 20, 21. Figure S3: Metaphase cells of sweet potato cultivars with the number of 5S rDNA is 5, and 7.
