# Supplementary material for: Comparative Chromosomal Localization of 45S and 5S rDNA Sites in 76 Purple-Fleshed Sweet Potato Cultivars
Source: Plants (Basel). 2020 Jul 8;9(7):865. doi: 10.3390/plants9070865 (PMC7412053; doi:10.3390/plants9070865)
Supplement: Supplementary file 1 [file plants-09-00865-s001.pdf]

S1 Metaphase cells of aneuploids

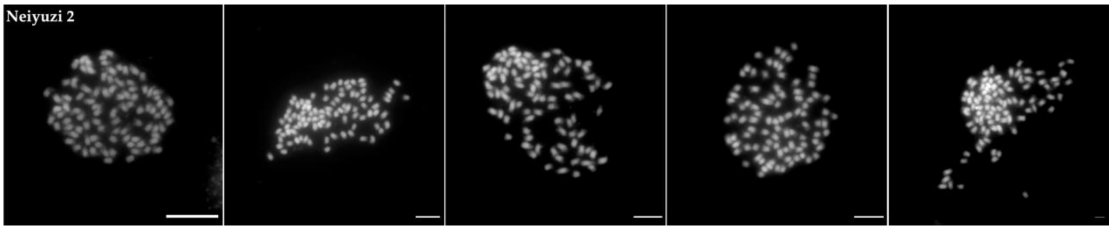

Figure 1 Metaphase cells of "Neiyuzi 2" showing 92 chromosomes. Scale bars, 5 μm.

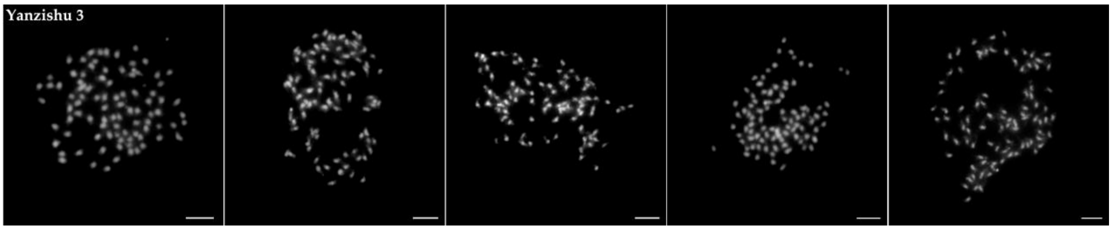

Figure 2 Metaphase cells of "Yanzishu 3" showing 89 chromosomes. Scale bars, 5 μm.

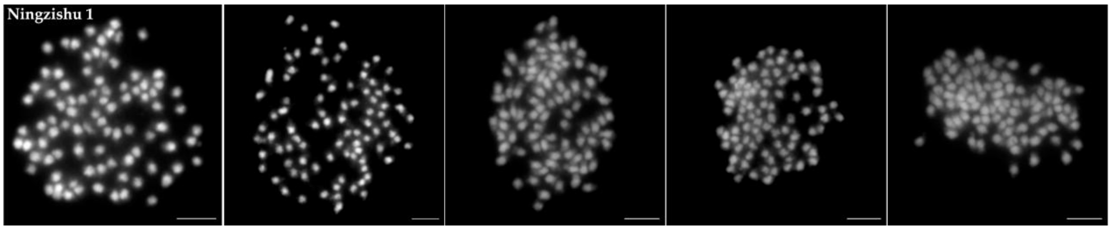

Figure 3 Metaphase cells of "Ningzishu 1" showing 88 chromosomes. Scale bars, 5 μm.

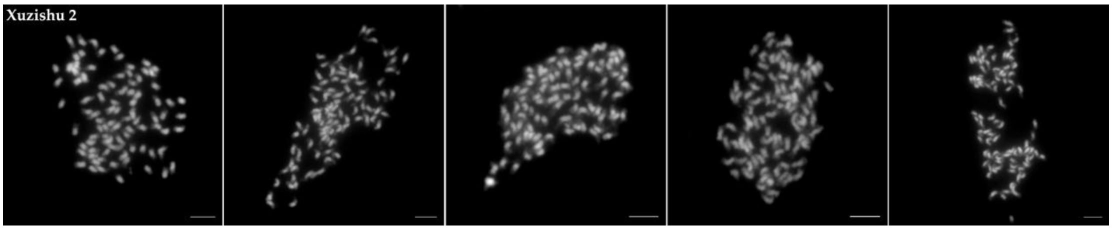

Figure 4 Metaphase cells of "Xuzishu 2" showing 88 chromosomes. Scale bars, 5 μm.

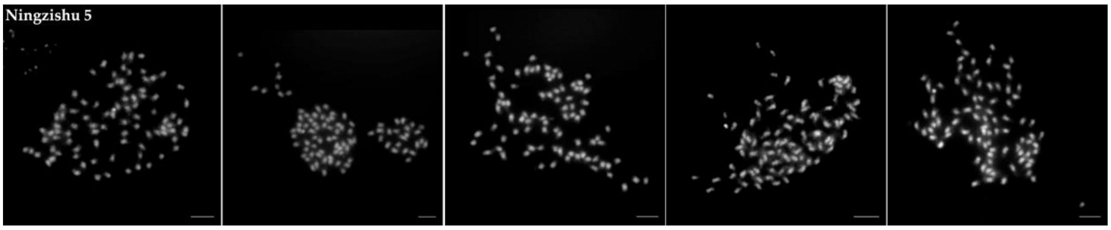

Figure 5 Metaphase cells of "Ningzishu 5" showing 89 chromosomes. Scale bars, 5 μm.

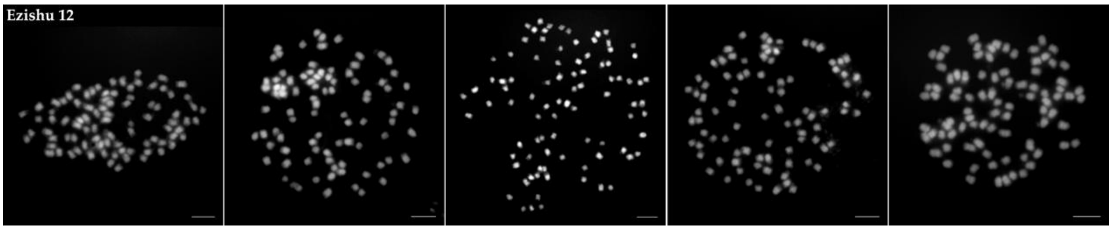

Figure 6 Metaphase cells of "Ezishu 12" showing 89 chromosomes. Scale bars, 5 μm.

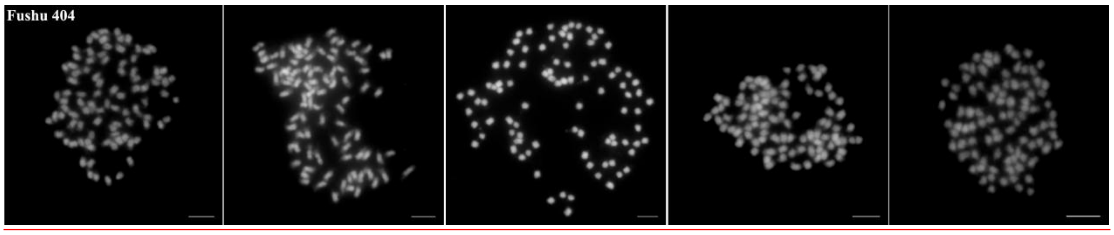

Figure 7 Metaphase cells of "Fushu 404" showing 92 chromosomes. Scale bars, 5 μm.

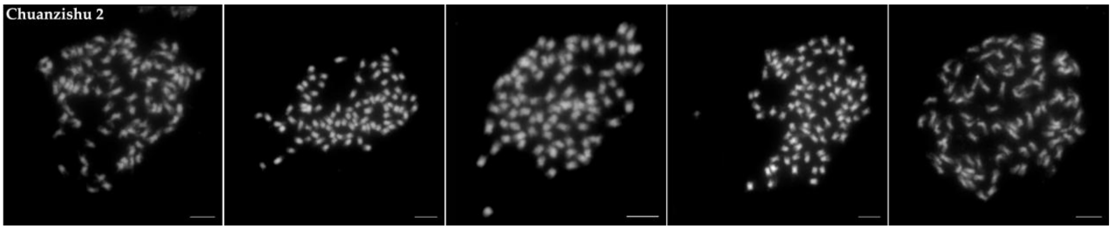

Figure 8 Metaphase cells of "Chuanzishu 2" showing 88 chromosomes. Scale bars, 5 μm.

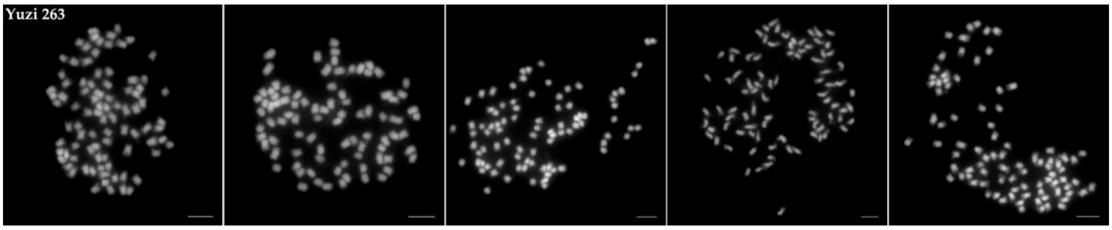

Figure 9 Metaphase cells of "Yuzi 263" showing 88 chromosomes. Scale bars, 5 μm.

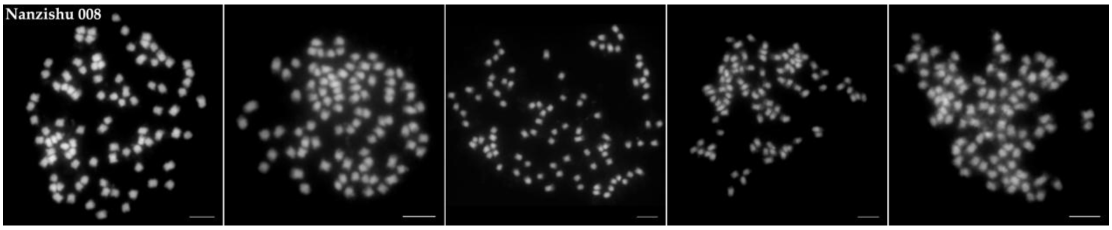

Figure 10 Metaphase cells of "Nanzishu 008" showing 91 chromosomes. Scale bars, 5 μm.

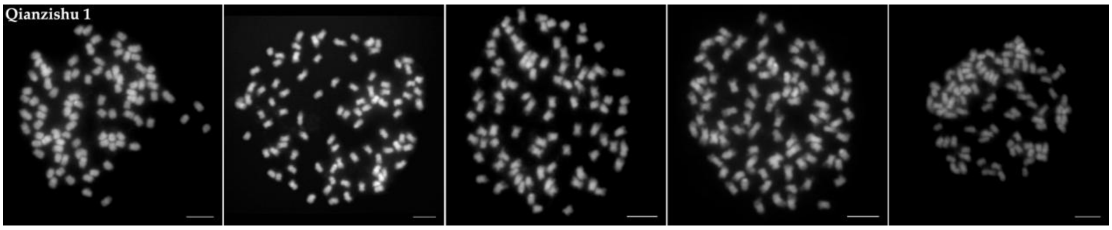

Figure 11 Metaphase cells of "Qianzishu 1" showing 91 chromosomes. Scale bars, 5 μm.

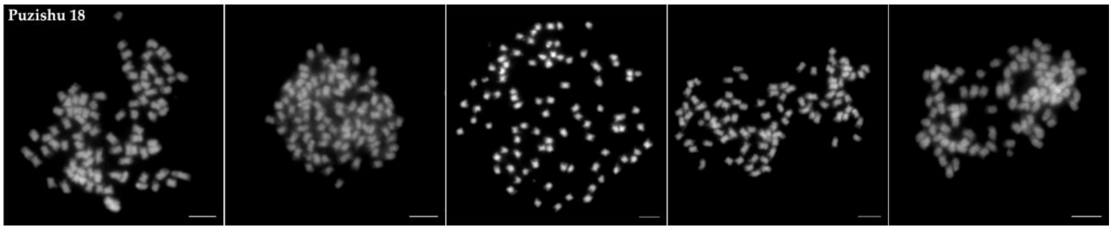

Figure 12 Metaphase cells of " Puzishu 18" showing 89 chromosomes. Scale bars, 5 μm.

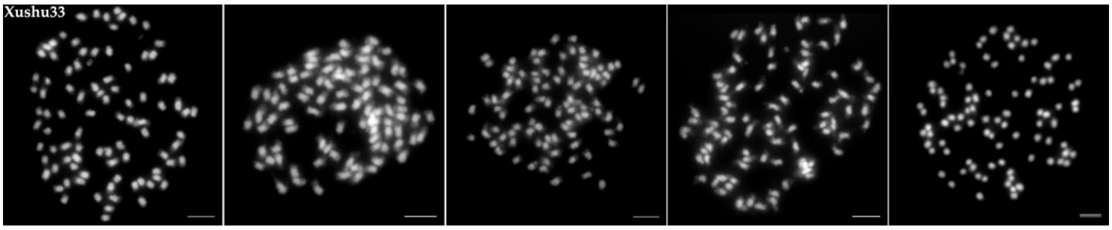

Figure 13 Metaphase cells of " Xushu33" showing 91 chromosomes. Scale bars, 5 μm.

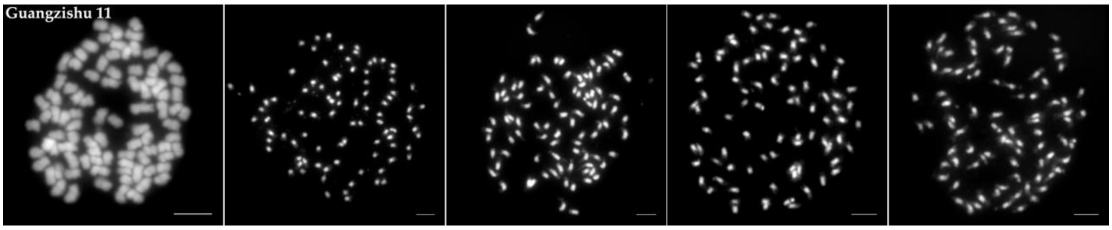

Figure 14 Metaphase cells of " Guangzishu 11" showing 89 chromosomes. Scale bars, 5 μm.

S2 Metaphase cells of sweet potato cultivars with the number of 45S rDNA is 16, 17, 19, 20, 21.

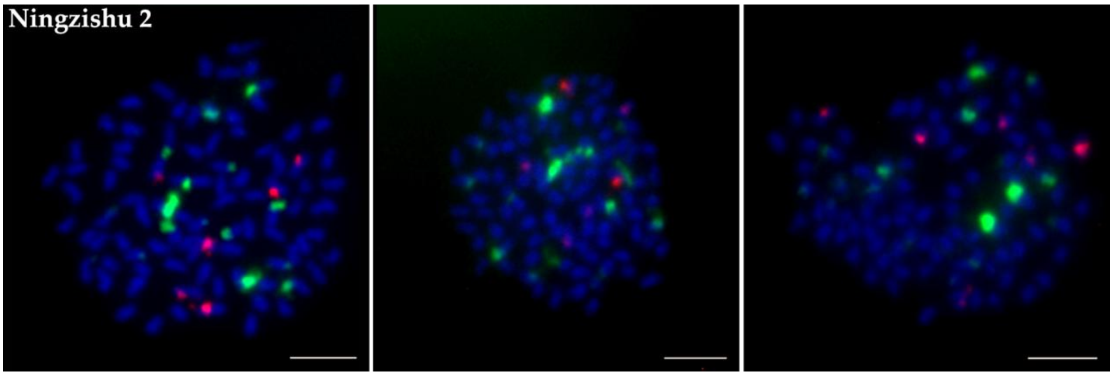

Figure 1 Metaphase cells of " Ningzishu 2" with the number of 45S rDNA is 16

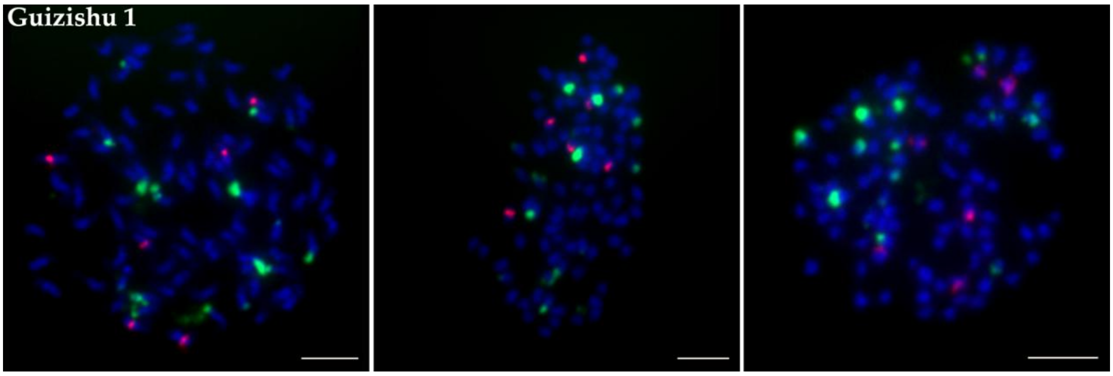

Figure 2 Metaphase cells of " Guizishu 1" with the number of 45S rDNA is 16

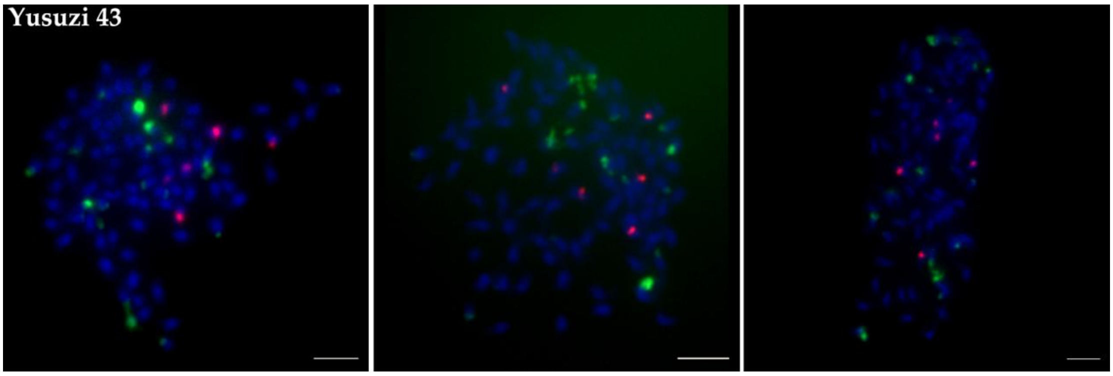

Figure 3 Metaphase cells of " Yusuzi 43" with the number of 45S rDNA is 16

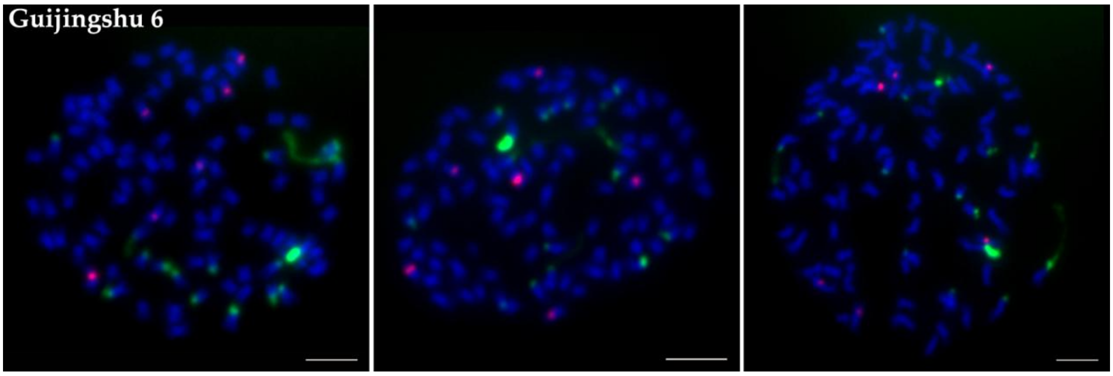

Figure 4 Metaphase cells of " Guijingshu 6" with the number of 45S rDNA is 16

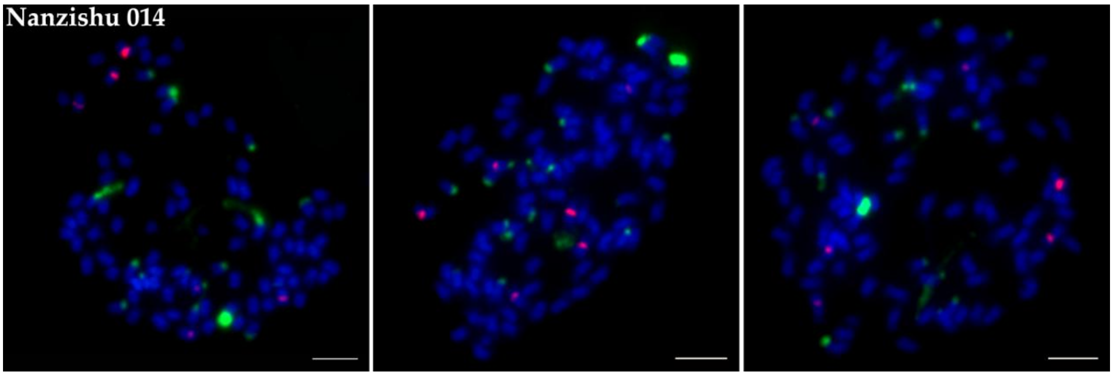

Figure 5 Metaphase cells of " Nanzishu 014" with the number of 45S rDNA is 16

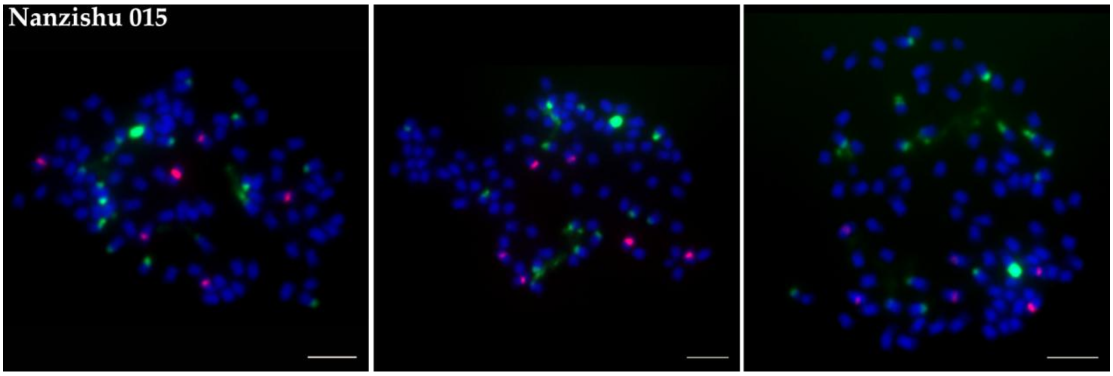

Figure 6 Metaphase cells of " Nanzishu 015" with the number of 45S rDNA is 16

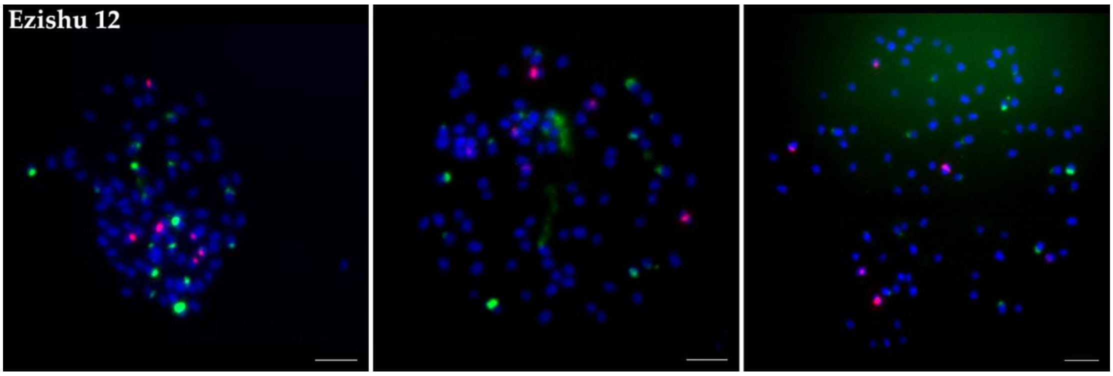

Figure 7 Metaphase cells of "Ezishu 12" with the number of 45S rDNA is 17

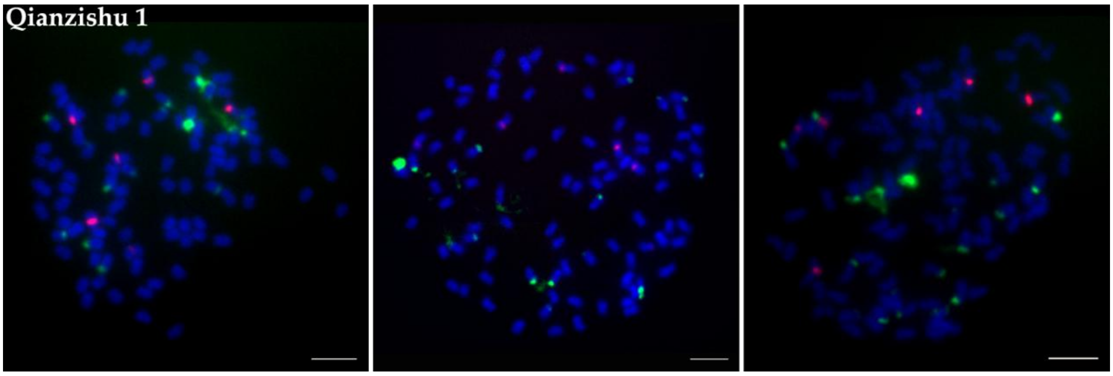

Figure 8 Metaphase cells of "Qianzishu 1" with the number of 45S rDNA is 17

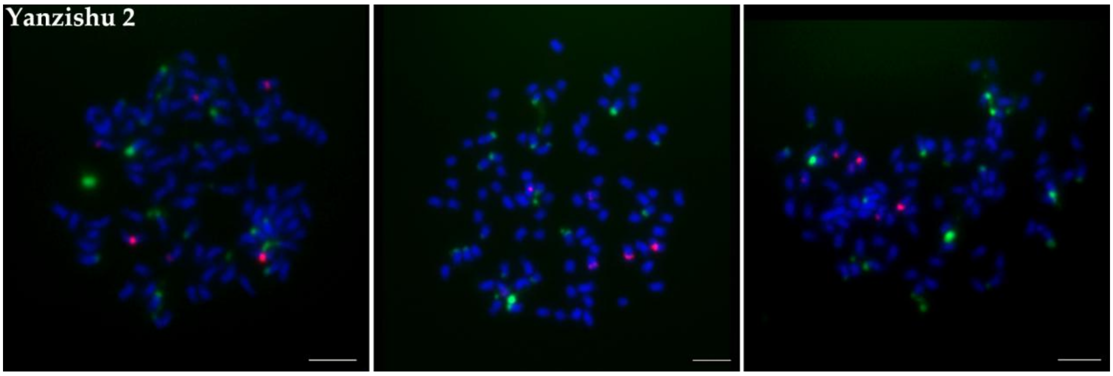

Figure 9 Metaphase cells of "Yanzishu 2" with the number of 45S rDNA is 19

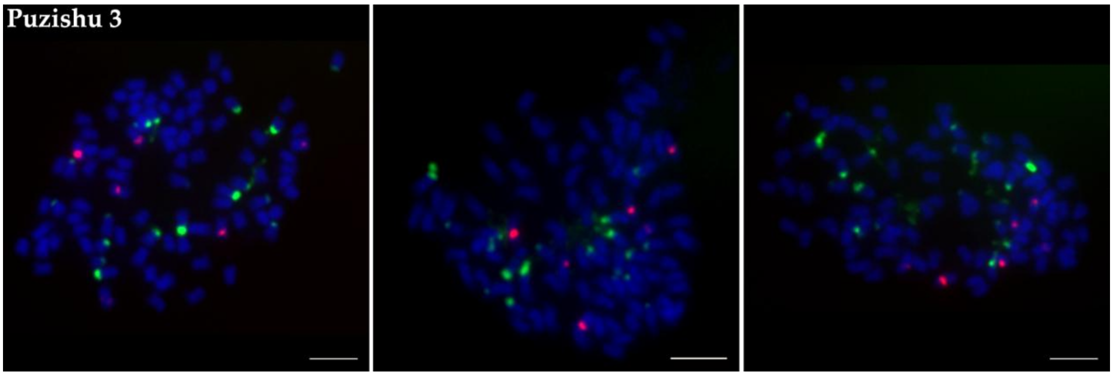

Figure 10 Metaphase cells of "Puzishu 3" with the number of 45S rDNA is 19

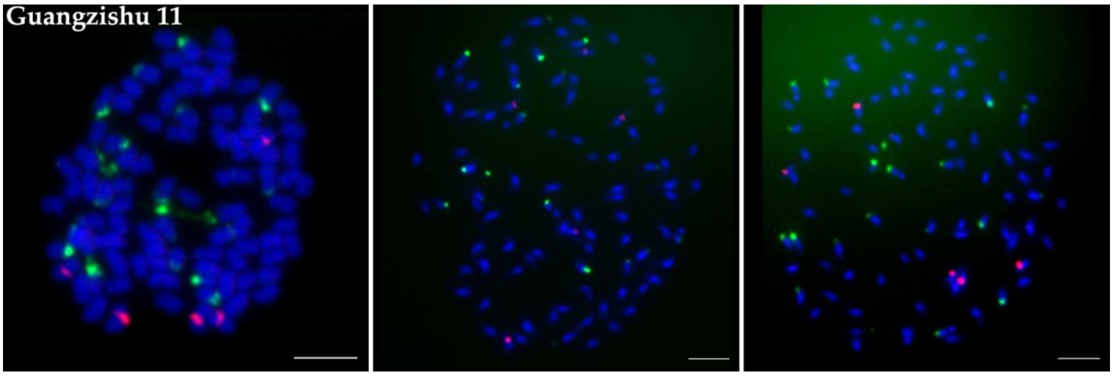

Figure 11 Metaphase cells of "Guangzishu 11" with the number of 45S rDNA is 19

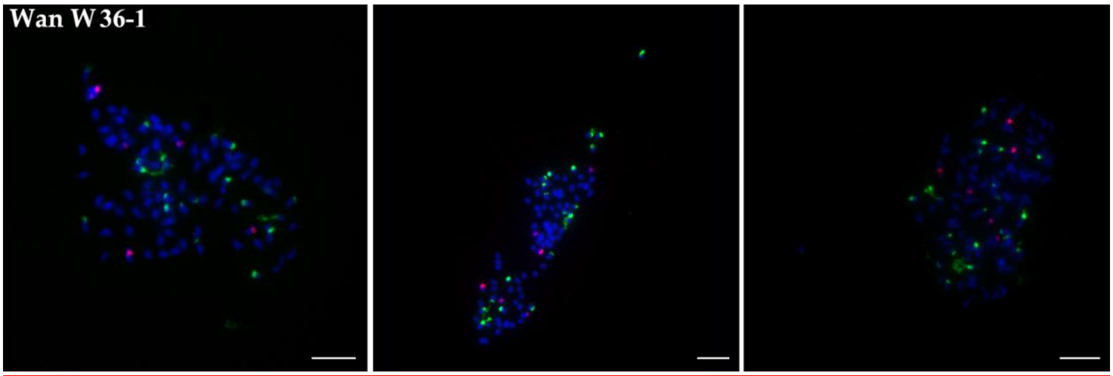

Figure 12 Metaphase cells of "Wan W36-1" with the number of 45S rDNA is 20

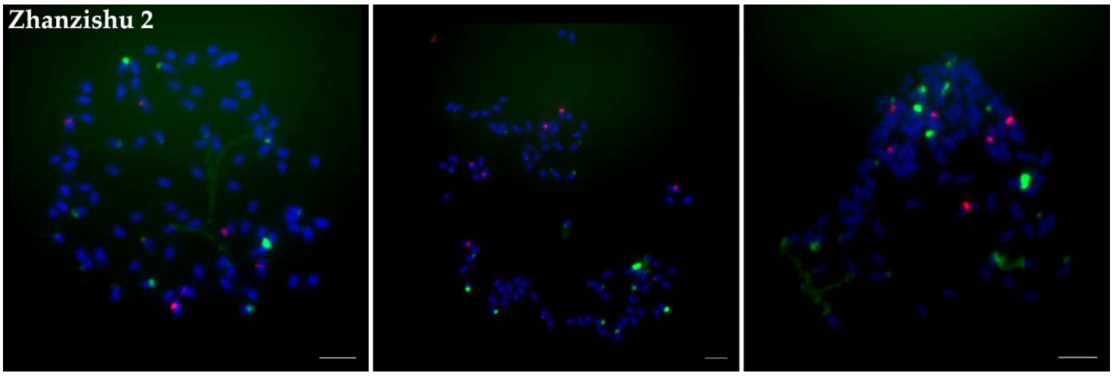

Figure 13 Metaphase cells of "Zhanzishu 2" with the number of 45S rDNA is 20

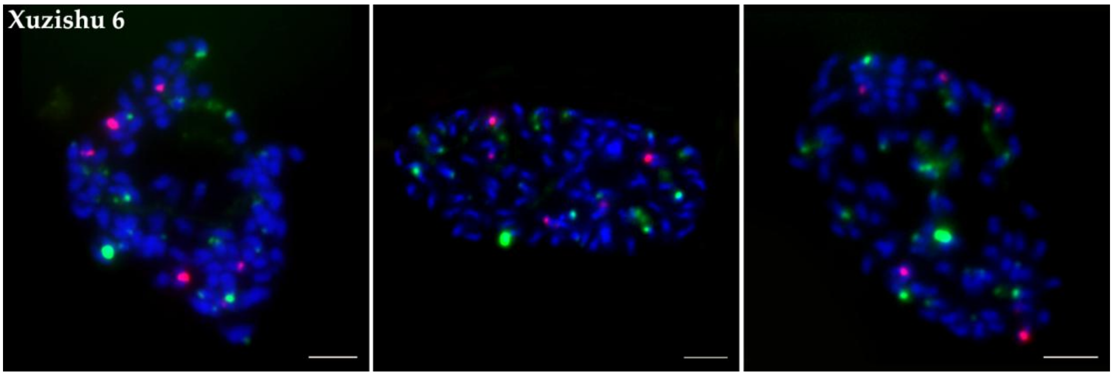

Figure 14 Metaphase cells of "Xuzishu 6" with the number of 45S rDNA is 20

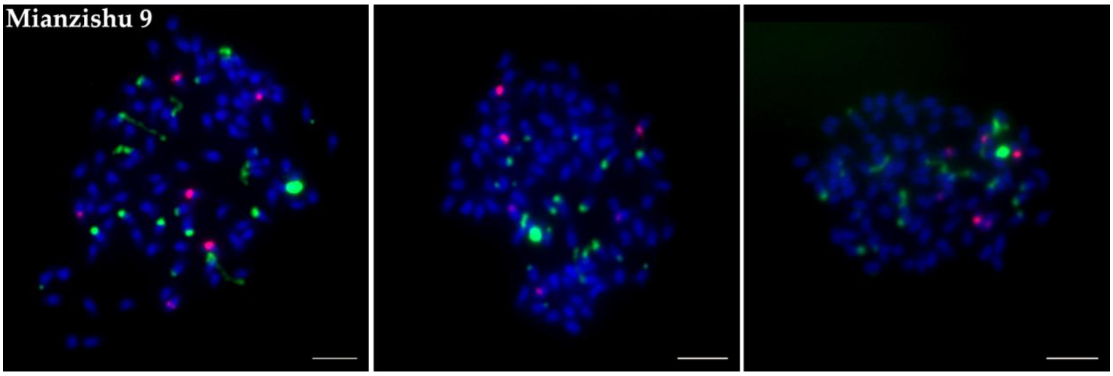

Figure 15 Metaphase cells of "Mianzishu 9" with the number of 45S rDNA is 20

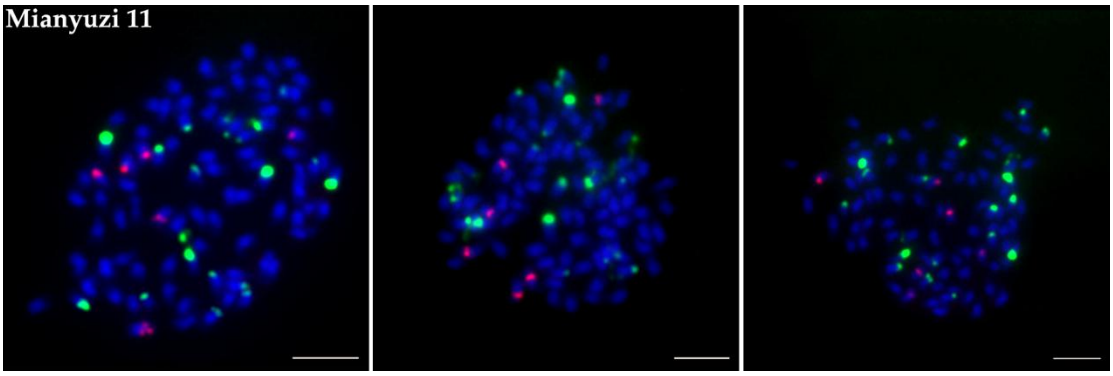

Figure 16 Metaphase cells of "Mianyuzi 11" with the number of 45S rDNA is 20

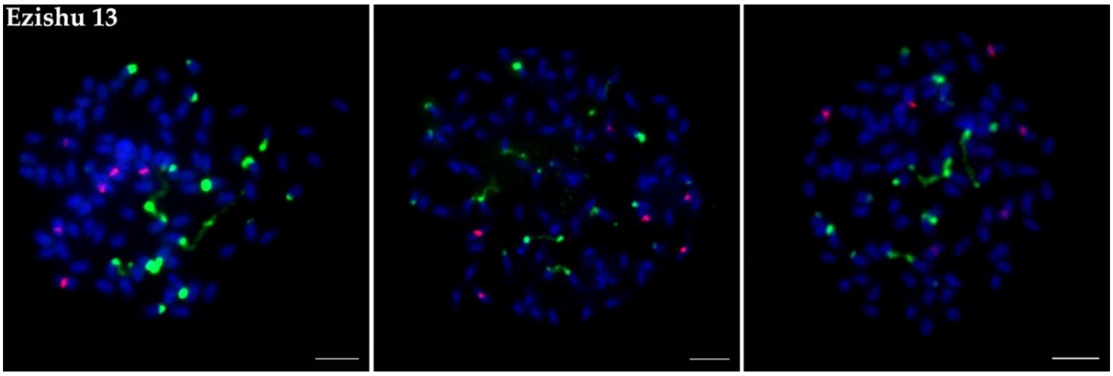

Figure 17 Metaphase cells of "Ezishu 13" with the number of 45S rDNA is 20

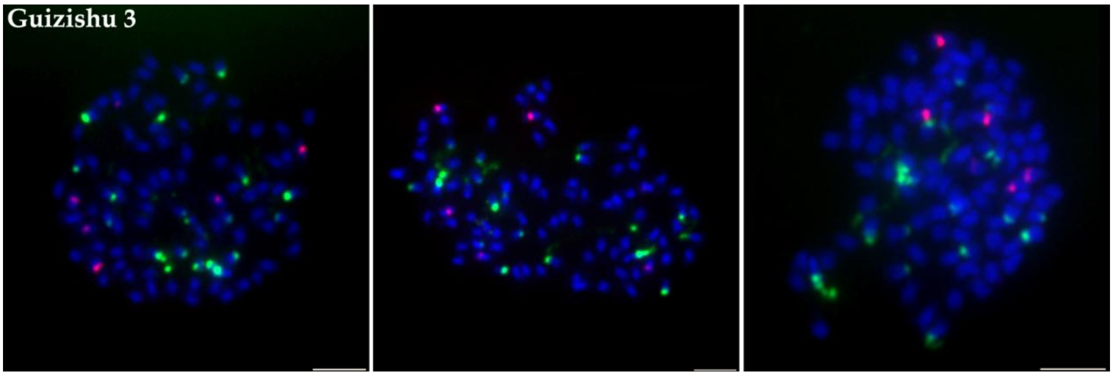

Figure 18 Metaphase cells of "Guizishu 3" with the number of 45S rDNA is 20

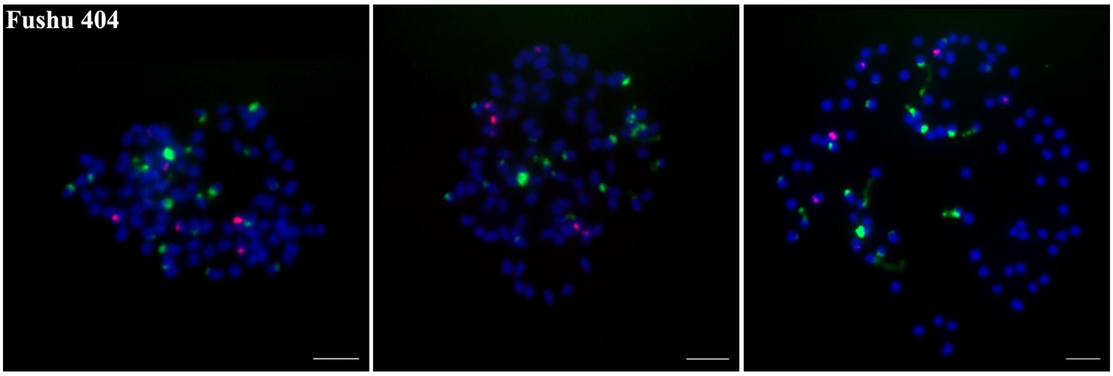

Figure 19 Metaphase cells of "Fushu 404" with the number of 45S rDNA is 20

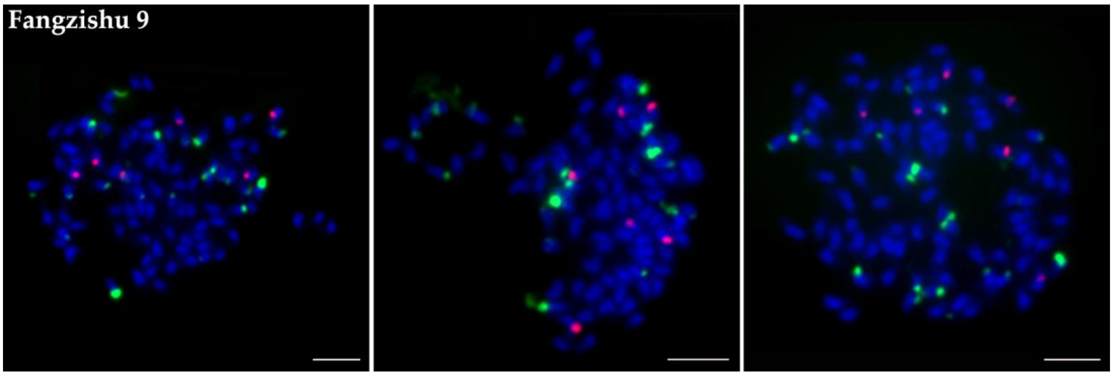

Figure 20 Metaphase cells of "Fangzishu 9" with the number of 45S rDNA is 20

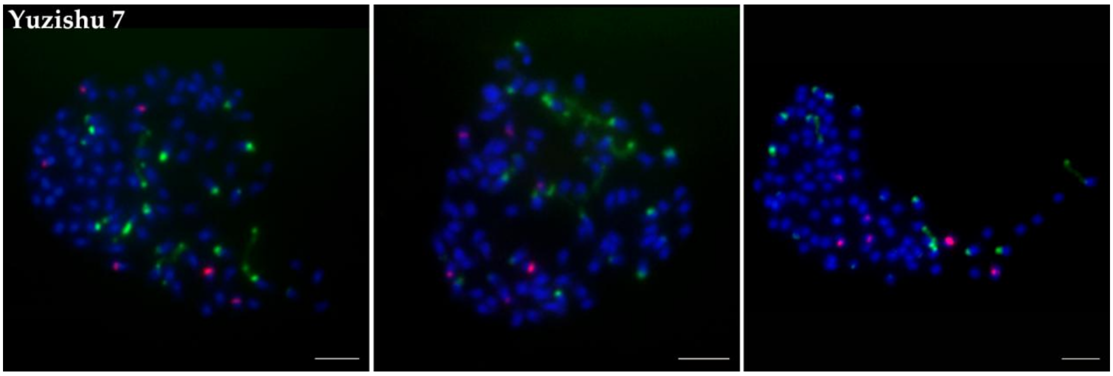

Figure 21 Metaphase cells of "Yuzishu 7" with the number of 45S rDNA is 20

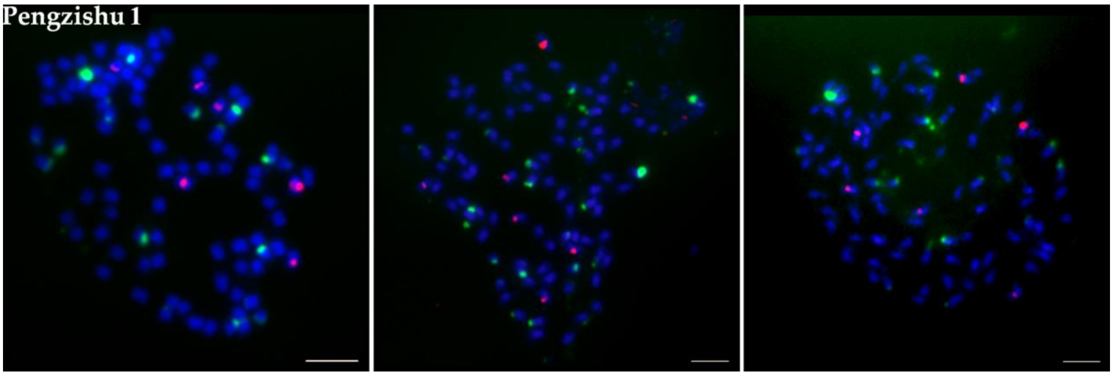

Figure 22 Metaphase cells of "Pengzishu 1" with the number of 45S rDNA is 20

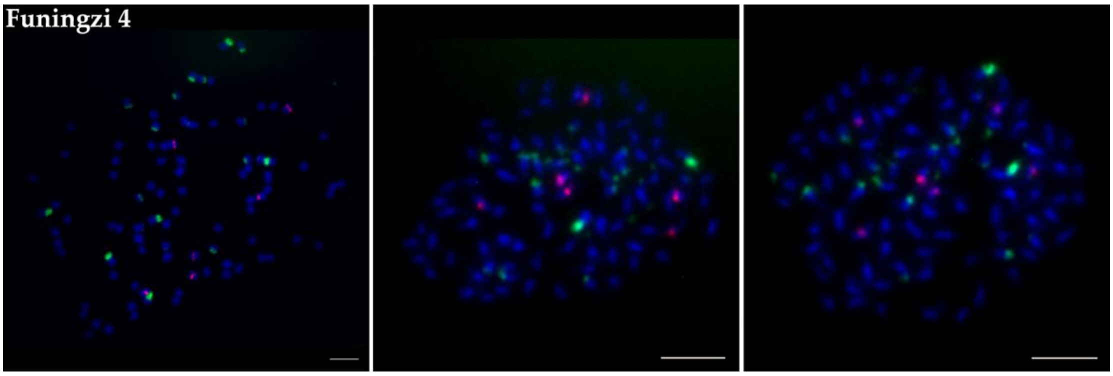

Figure 23 Metaphase cells of "Funingzi 4" with the number of 45S rDNA is 20

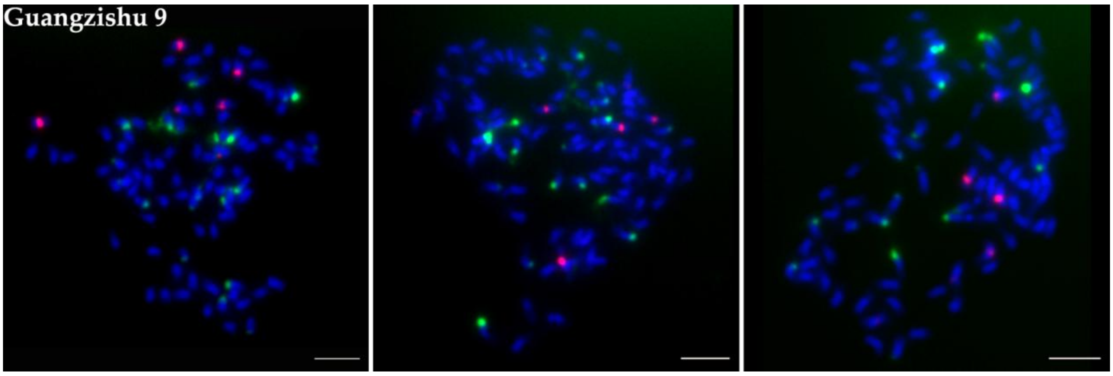

Figure 24 Metaphase cells of "Guangzishu 9" with the number of 45S rDNA is 20

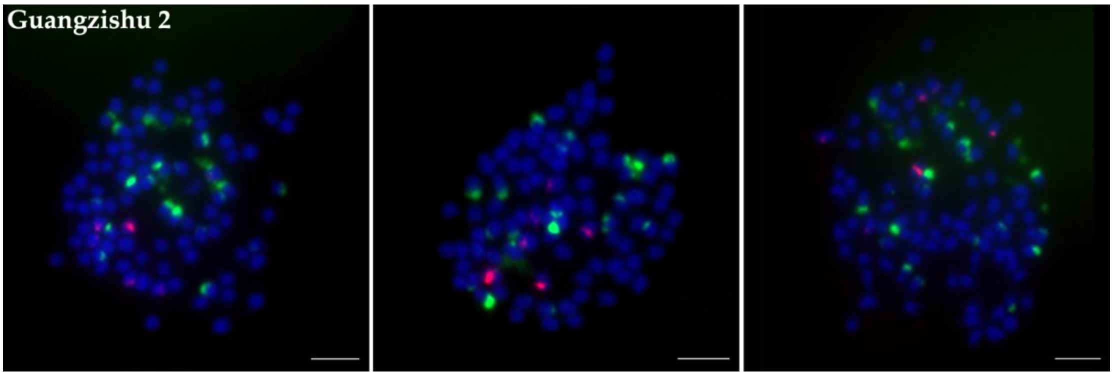

Figure 25 Metaphase cells of "Guangzishu 2" with the number of 45S rDNA is 20

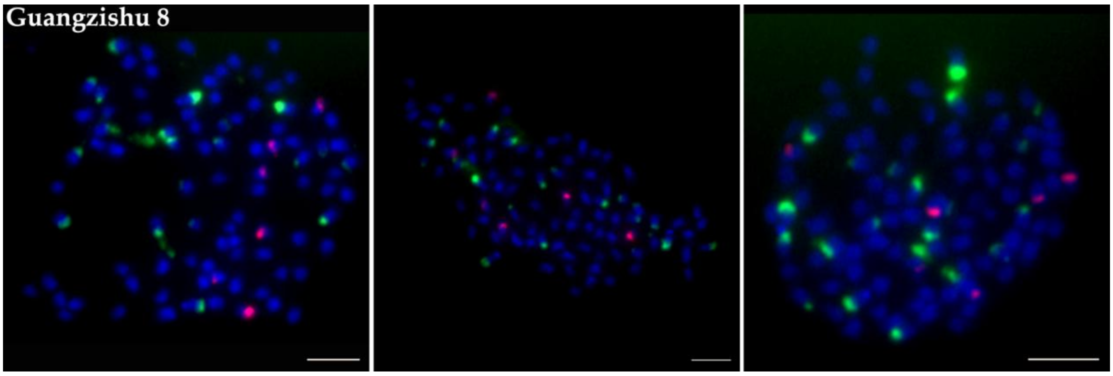

Figure 26 Metaphase cells of "Guangzishu 8" with the number of 45S rDNA is 20

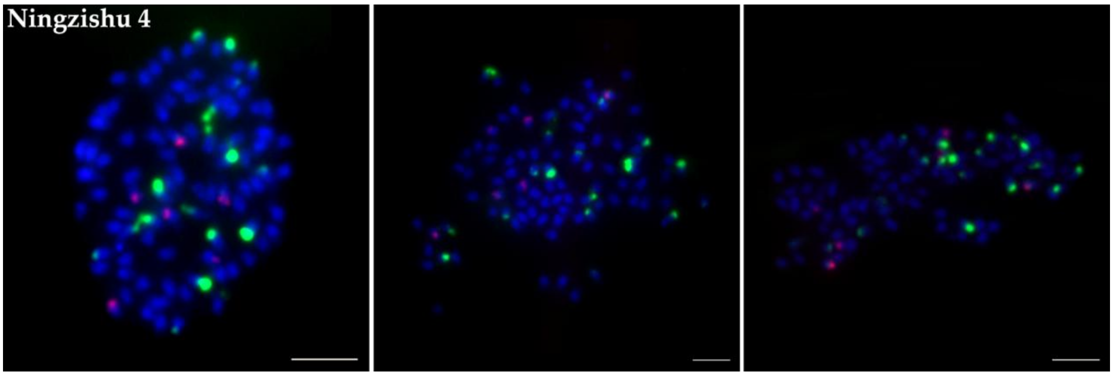

Figure 27 Metaphase cells of "Ningzishu 4" with the number of 45S rDNA is 21

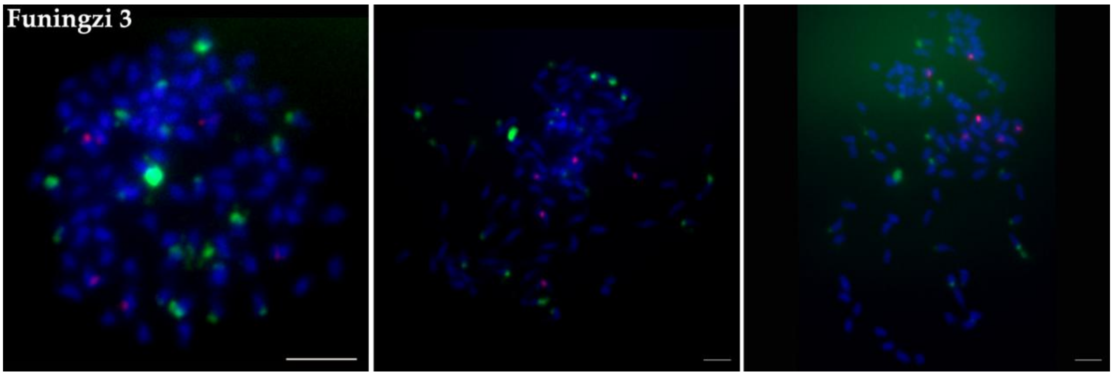

Figure 28 Metaphase cells of "Funingzi 3" with the number of 45S rDNA is 21

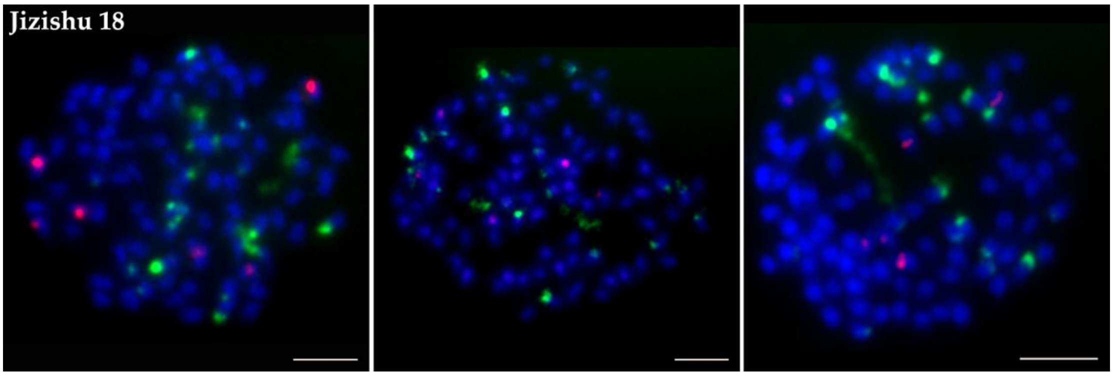

Figure 29 Metaphase cells of "Jizishu 18" with the number of 45S rDNA is 21

S3 Metaphase cells of sweet potato cultivars with the number of 5S rDNA is 5, and 7.

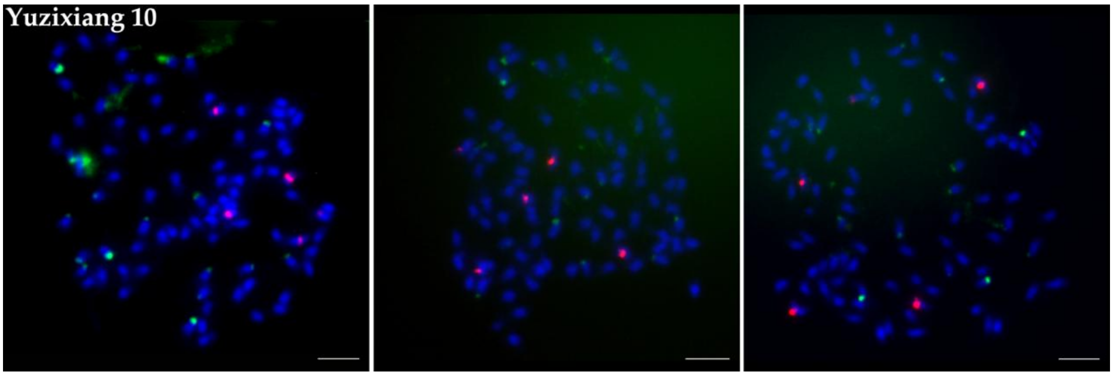

Figure 1 Metaphase cells of "Yuzixiang 10" with the number of 5S rDNA is 5

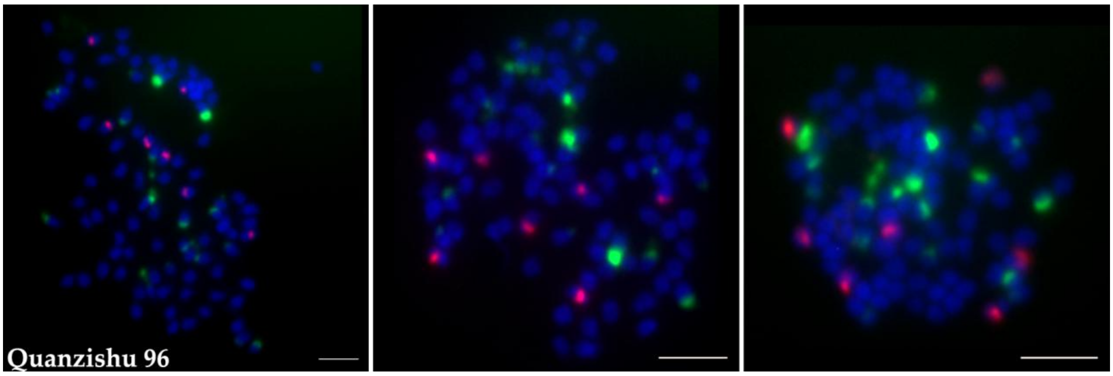

Figure 2 Metaphase cells of "Quanzishu 96" with the number of 5S rDNA is 7
